# Inhibition of constitutively active Jak-Stat pathway suppresses cell growth of human T-cell leukemia virus type 1-infected T-cell lines and primary adult T-cell leukemia cells

**DOI:** 10.1186/1742-4690-8-1

**Published:** 2011-01-06

**Authors:** Mariko Tomita, Hirochika Kawakami, Jun-nosuke Uchihara, Taeko Okudaira, Masato Masuda, Takehiro Matsuda, Yuetsu Tanaka, Kazuiku Ohshiro, Naoki Mori

**Affiliations:** 1 Division of Molecular Virology and Oncology, Graduate School of Medicine, University of the Ryukyus, 207 Uehara, Nishihara, Okinawa 903-0215, Japan; 2 Division of Endocrinology and Metabolism, Faculty of Medicine, University of the Ryukyus, 207 Uehara, Nishihara, Okinawa 903-0215, Japan; 3 Division of Child Health and Welfare, Faculty of Medicine, University of the Ryukyus, 207 Uehara, Nishihara, Okinawa 903-0215, Japan; 4 Division of Immunology, Faculty of Medicine, University of the Ryukyus, 207 Uehara, Nishihara, Okinawa 903-0215, Japan; 5 Department of Internal Medicine, Naha Prefectural Hospital, 1-3-1 Yogi, Naha, Okinawa 902-8531, Japan

## Retraction

The authors of this article [1] would like to retract it. It contains duplicated Tax and actin Western blotting images. These misrepresentations do not change the scientific conclusion of the paper. The first author, Mariko Tomita and last author, Naoki Mori, take full responsibility for the duplication and misrepresentation of the figures in this paper, state that none of the co-authors were involved in or aware of these events, and apologizes to the readers, reviewers and editors of *Retrovirology *for publishing these duplicated images.
